# Lateral Bowing of Femur Associated With Older Age, Shorter Stature, and Lower Bone Mineral Density

**DOI:** 10.7759/cureus.19735

**Published:** 2021-11-19

**Authors:** Yasuhiro Furihata, Tetsuhiro Ishikawa, Joe Katsuragi, Takanori Omae, Yasuhito Sasaki, Tomotaka Umimura, Ryutaro Iwasaki, Ryutaro Shingyouuchi, Susumu Tashiro, Michitaka Namiki, Seiji Ohtori

**Affiliations:** 1 Orthopaedic Surgery, Sanmu Medical Center, Sanmu, JPN; 2 Orthopaedic Surgery, Midorinoha Yoh Memorial Hospital, Chiba, JPN; 3 Orthopaedics, Sanmu Medical Center, Sanmu, JPN; 4 Orthopaedics, Chiba University Hospital, Chiba, JPN; 5 Department of Orthopaedic Surgery, Graduate School of Medicine, Chiba University, Chiba, JPN

**Keywords:** osteoporosis, femoral bowing, osteoarthritis (oa), dxa, bone mineral density

## Abstract

We often encounter elderly patients with femur bowing. According to literature, femoral bowing is correlated with patient characteristics such as aging, race, atypical femoral fracture (AFF), and osteoporosis. However, the clear relationships between these factors and femoral bowing are still unknown. In addition, most previous reports have been based only on X-rays and may not provide accurate information due to femur rotation and inter-operator reliability when compared to the information obtained using computed tomography (CT) scans. The purpose of this study was to examine the factors associated with anterior and lateral bowing in detail, by using three-dimensional preoperative measurement software Zed Hip®︎ (LEXI Co. Ltd., Tokyo, Japan). A total of 364 patients with trochanteric hip or femoral neck fractures, or osteoarthritis, treated in our hospital were included in this study. Of these, 61 patients older than 50 years, who had complete CT volume data for the entire length of the femur on the healthy side and bone mineral density (BMD) measured by trunk dual-energy X-ray absorptiometry (DXA), were investigated. There were 13 males and 48 females, aged 53-97 years (mean 78.7±10.8 years). We defined the starting and ending points of the femoral diaphysis to measure anterior bowing (AB) and lateral bowing (LB) of the femoral diaphysis. The correlation between AB or LB with each patient's characteristics (age, height, weight, lumbar BMD, and femoral BMD) was examined retrospectively. AB did not correlate with any of the patient parameters. LB weakly positively correlated with age and was negatively correlated with height and femoral (greater trochanter) bone density. Weight was in no correlation with either AB or LB. A novel three-dimensional approach was used for measurements that may be more accurate than plain two-dimensional radiographs.

## Introduction

In daily practice, we often see anterior and lateral bowing of the femur [1]. The bowing of the femur might affect the surgical plan and outcomes in cases requiring internal fixation surgery for fractures [2,3], or implant insertion for hip and knee arthroplasty [4,5]. Several reports have shown that femoral bowing is correlated with patient characteristics such as aging, race, osteoarthritis, and osteoporosis [1,6-10]. Zyang et al. reported that the extent of the bowing progresses with aging [11]. Papaioannou et al. showed a correlation between femoral bowing and osteoporosis [12]. Other reports have suggested that the greater the anterolateral curvature, the more likely is the occurrence of an atypical femoral fracture (AFF) [12-16]. However, the complex relationships between patient characteristics and femoral bowing are still unknown. In addition, most previous reports have been based only on X-rays that may not provide as accurate antero-posterior (AP) or lateral images, depending on the patient's position, compared to examinations done using computed tomography (CT) scans [2,3,8,11,17,18]. The purpose of this study was to examine the factors associated with anterior and lateral bowing in detail by using three-dimensional preoperative measurement software Zed Hip®︎ (LEXI Co. Ltd., Tokyo, Japan). We measured femoral bowing of the contralateral side in patients with proximal femoral fracture or osteoarthritis to investigate the relationship with various patient characteristics.

## Materials and methods

A total of 364 patients with trochanteric hip or femoral neck fractures, or osteoarthritis, treated in our hospital during February 2019-November 2020 were included in this study. Of these, 61 cases older than 50 years, except for high energy trauma, who had complete CT volume data for the entire length of the femur on the healthy side, and bone mineral density (BMD) measured by trunk bone dual-energy X-ray absorptiometry (DXA), were investigated. There were 13 males and 48 females, aged 53-97 years (mean 78.7±10.8 years). Informed consent was obtained from all participants in this study. This study was approved by the institutional review board of the authors’ affiliated institutions (the ethics committee of Sanmu Medical Center, 2020-007).

There is no clear definition of the boundaries of femoral diaphysis [17]. Therefore, we defined the starting and ending points of the femoral diaphysis based on specific morphological points to measure anterior and lateral bowing of the femoral diaphysis. Zed Hip®︎, a three-dimensional preoperative planning software, was used for the measurements. The CT data of the entire length of the femur on the healthy side was imported into the software, and the center of the femoral head, medial epicondyle, lateral epicondyle, the most posterior points of the medial condyle, and lateral condyle of the femur were plotted with three-dimensional spatial coordinates to project the femur with a defined frontal surface in three-dimensional space. 

**Figure 1:**
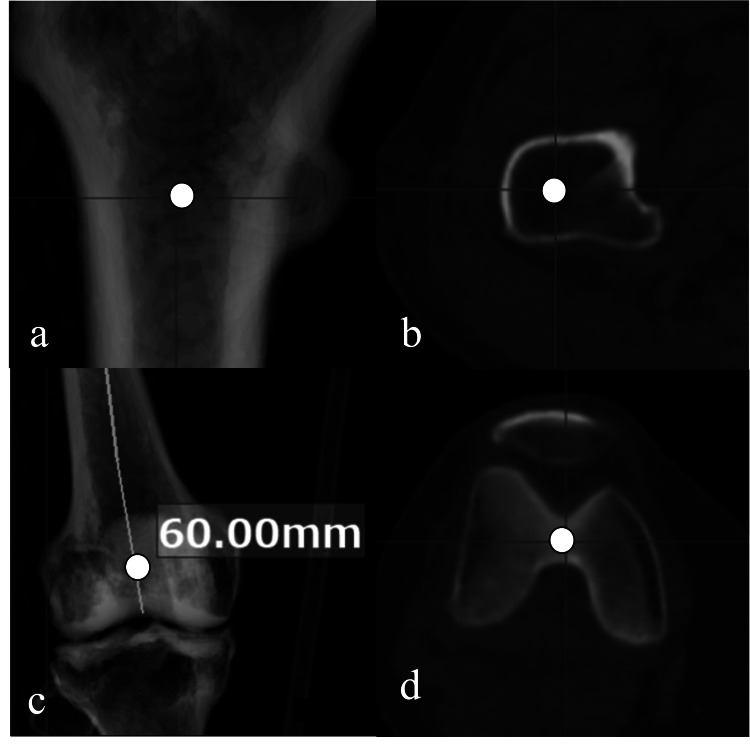
Defining the reference axis on the three-dimensional preoperative planning software The most proximal starting point (a: coronal section, b: axial section). The most distal endpoint (c: coronal section, d: axial section).

We eliminated parts of the femur superior to the lesser trochanter, and the distal femoral condyles, which are not directly related to bowing of the femoral diaphysis. The most proximal starting point was the center of the medullary cavity at the level of the apex of the lesser trochanter, as that point is fixed and easily defined for all subjects (Figures 1a, 1b). Similarly, the most distal endpoint was the beginning of the distal femoral anatomical axis, in other words, the center of the medullary cavity at a height of 60 mm proximal to the distal femoral condylar groove as defined by Itokawa et al. (Figures 1c, 1d) [19]. Each point can be determined as only one on the CT.

**Figure 2:**
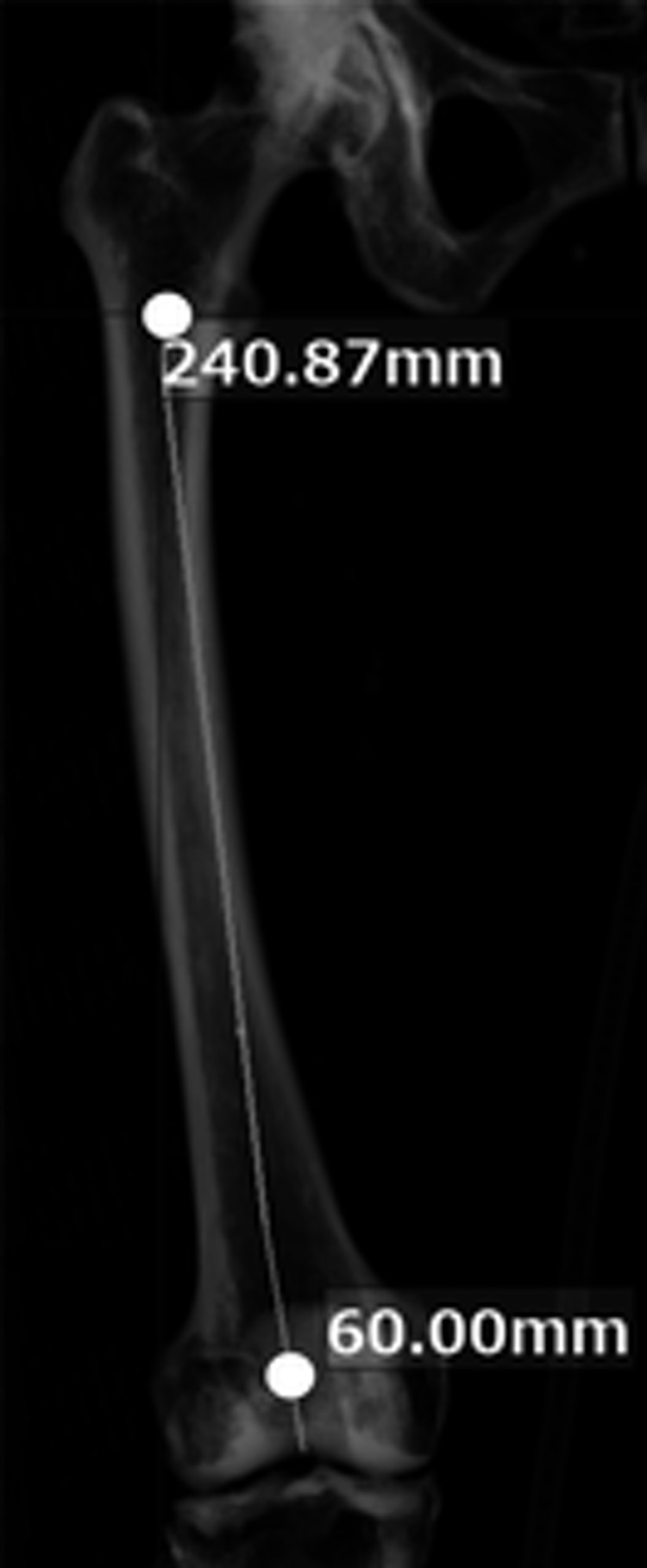
Setting the reference axis The line connecting two points (white circles in the image) was set as the reference axis.

The center of the medullary cavity was defined as 60 mm proximal to the inferior end of the femoral pulley groove and was connected to the center of the medullary cavity at the level of the apex of the lesser trochanter. This line segment was defined as the reference axis, and its length was set as the reference axis length (Figure 2).

**Figure 3:**
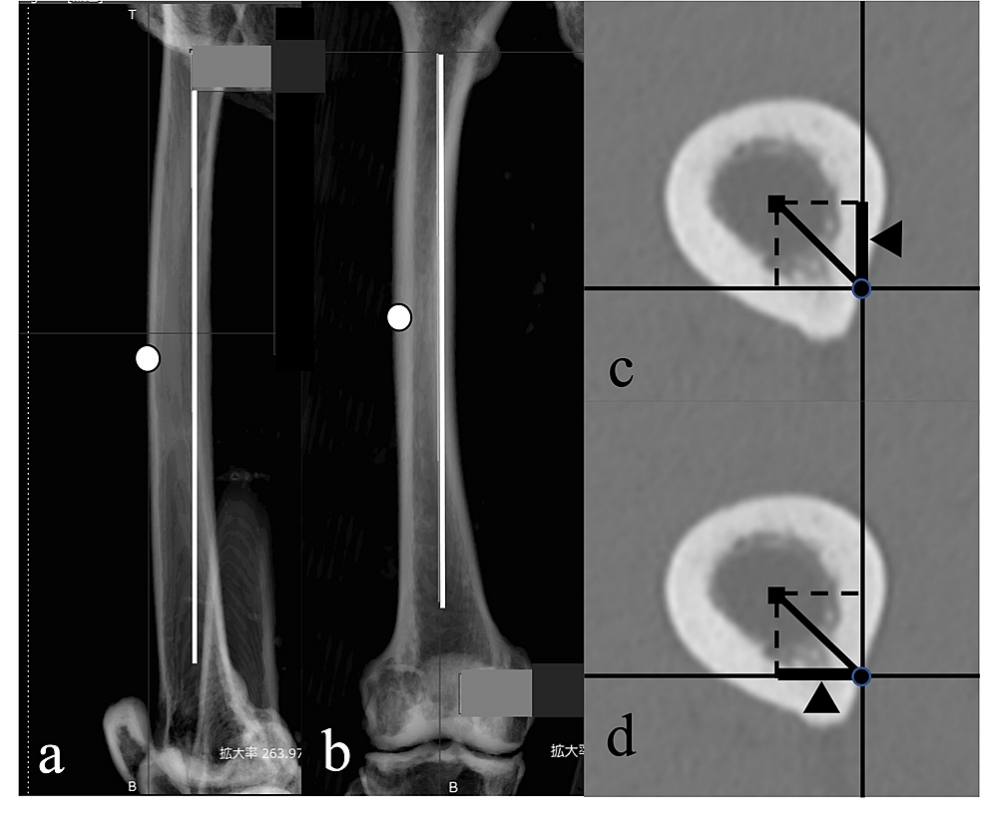
Determining the point of maximum anterior bowing and lateral bowing a: In the lateral image, the farthest point on the cortical bone from the reference axis was determined to be the point of maximum anterior bowing. b: In the AP image, the farthest point on the cortical bone from the reference axis was determined to be the point of maximum lateral bowing. c: In the axial plane, the anterior bowing length is the sine of the distance between the center of the bone marrow cavity and the reference axis at the point of maximum anterior bowing. d: In the axial plane, the lateral bowing length is the cosine of the distance between the center of the bone marrow cavity and the reference axis at the point of maximum lateral bowing.

In the sagittal midline reconstruction image rotated 90° from the AP view, the point of the femoral cortex farthest from the reference axis was defined as the point of maximum anterior bowing (Figure 3a). Using the same method, the distance in the frontal image between the center of the medullary cavity at the level of the outermost lateral bowing point (Figure 3b). The sine of the center of the medullary cavity and the reference axis at the level of the most anterior bowing point, that is, the distance between the center of the medullary cavity and the reference axis, was defined as the anterior bowing length (Figure 3c). The cosine of those at the level of the most lateral bowing point was defined as the lateral bowing length as well (Figure 3d).

**Figure 4:**
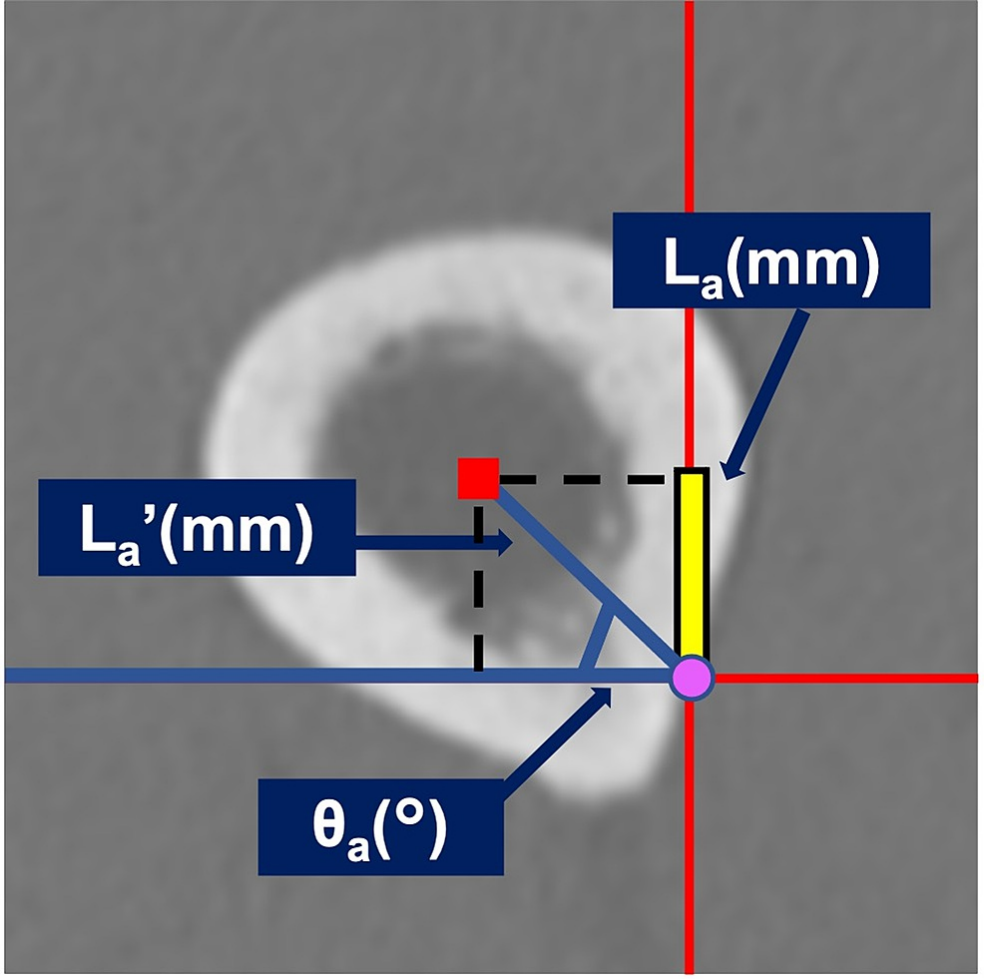
Measurement of AB and LB The axial plane of the femur at the point of maximum anterior bowing. Center of the medullary cavity (■) and the reference axis (●) at the level of the most anterior bowing point.

Each value was divided by the reference axis length to obtain a ratio for anterior bowing (AB) and lateral bowing (LB). The center of the medullary cavity (■) and the reference axis (●) at the level of the most anterior bowing point are shown in Figure 4.

The anterior bowing length (L_a_mm) was defined as 

L_a _＝L_a’_sin θ_a_

where L_a’_= the distance between the center of the medullary cavity and the reference axis (mm) and θ_a_ = the angle between the line segment connecting the center of the medullary cavity and the reference axis with the transverse axis (°).

Anterior bowing (AB) was defined as the value of L_a_ divided by the reference axis length L_f_, where AB= L_a_ / L_f_

Lateral bowing (LB) was defined as below in the same way.

L_l_ ＝L_l’_sin θ_l_

L_f_, where LB= L_l_ / L_f_

The correlation between anterior and lateral bowing and each patient's characteristics (age, height, weight, lumbar BMD, and femoral BMD) was examined by Pearson’s product-moment correlations.

## Results

**Figure 5:**
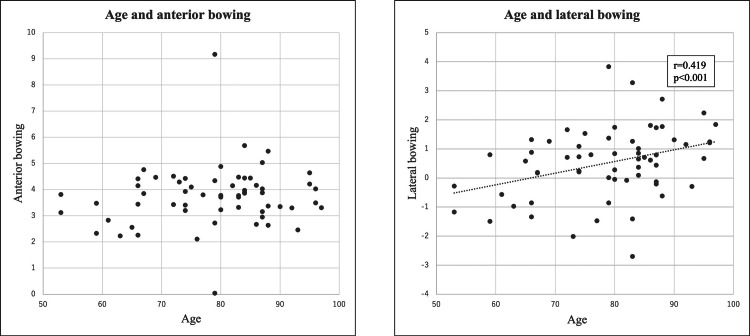
The scatter diagram between age and AB, LB respectively. There was no correlation between age and anterior bowing (AB). There was a positive correlation between age and lateral bowing (LB).

**Figure 6:**
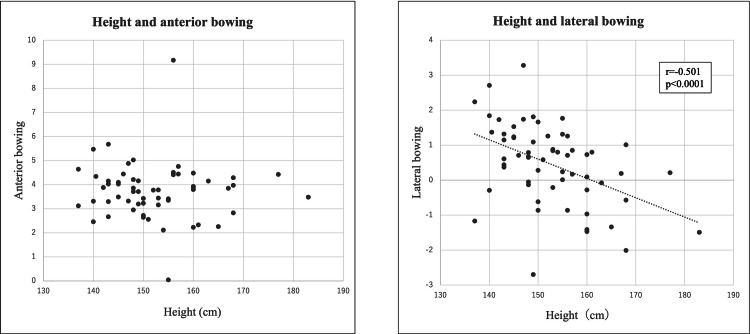
The scatter diagram between height and AB, LB respectively. There was no correlation between height and anterior bowing (AB). There was a negative correlation between height and lateral bowing (LB).

**Figure 7:**
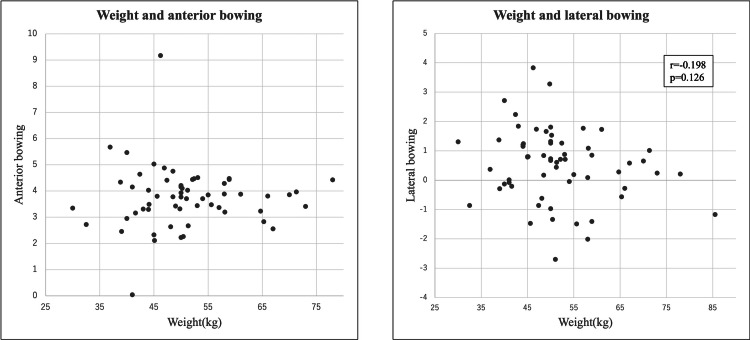
The scatter diagram between weight and AB, LB respectively. There was no correlation between weight and either anterior bowing (AB) or lateral bowing (LB).

**Figure 8:**
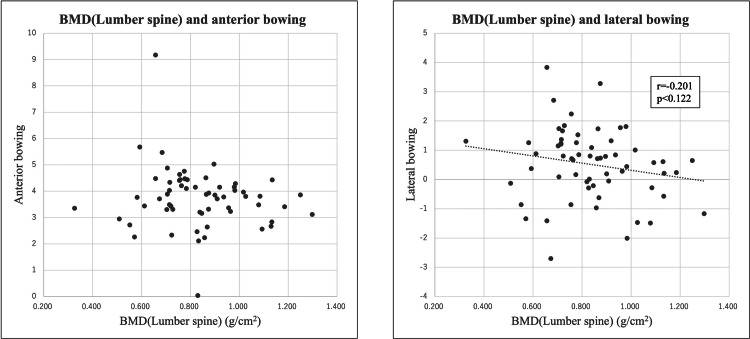
The scatter diagram between BMD (lumbar spine) and AB, LB respectively. There was no correlation between BMD (lumbar spine) and either anterior bowing (AB) or lateral bowing (LB). BMD = Bone mineral density

**Figure 9:**
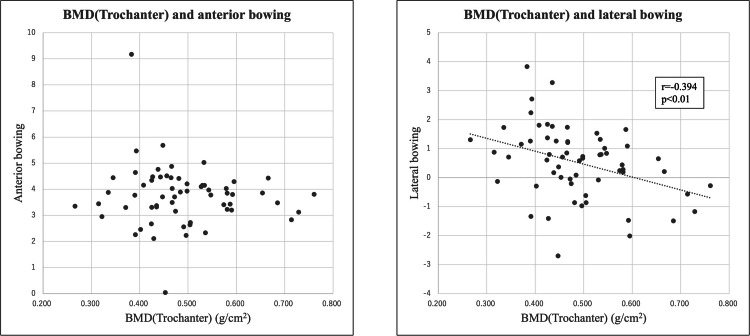
The scatter diagram between BMD (trochanteric) and AB, LB respectively. There was no correlation between BMD (trochanteric) and anterior bowing (AB). There was a negative correlation between BMD (trochanteric) and lateral bowing (LB). BMD = Bone mineral density

Averages of the 61 subjects’ characteristics were: age 78.7±10.8 years, height 152.6±9.4 cm, weight 51.53±10.6 Kg, lumbar spine BMD 0.837±0.187 g/cm^3^, and femoral BMD 0.489±0.102 g/cm^3^. The correlations between AB and LB with each patient's age, height, weight, lumbar BMD, and femoral BMD were determined retrospectively (Figures 5-9). There was no correlation between age and AB and a positive correlation (r=0.419, p<0.001) between age and LB (Figure 5).

There was no correlation between height and AB and a negative correlation (r=-0.501, p<0.0001) between height and LB (Figure 6). 

There was no correlation between weight and either AB or LB (Figure 7). 

There was no correlation between BMD (lumbar spine) and either AB or LB (Figure 8). 

There was no correlation between BMD (trochanteric) and AB and a negative correlation (r=-0.394, p<0.01) between BMD (trochanteric) and LB (Figure 9). 

There was no correlation between AB and any patient characteristic. There was a positive correlation between LB with age and negative correlation with height and a weak negative correlation with BMD of the greater trochanter and LB. No evident correlation was found between LB and either body weight or lumbar BMD. 

## Discussion

Three major results were obtained in this study. First, AB does not correlate with any of the patient parameters. Second, LB weakly positively correlates with age and negatively with height. Third, LB is weakly negatively correlated with femoral (greater trochanter) bone density.

It is suggested that there is a little individual variation of AB, regardless of the values of various parameters. There have been many reports on femoral AB suggesting that it has the same relationships to age and bone density as LB [1,11,16]. The current study does not support that conclusion. However, the results of these prior studies were all based on simple radiographic images. There have been few studies using CT except for reports related to prostheses. Simple radiographs are two-dimensional. Radiographs have the disadvantages of being prone to errors depending on the examiner and patient, and cannot discern the three-dimensional torsion of the femur, which can cause errors [1,17]. On the other hand, in our study, since the frontal plane was defined and the reference axis was set by the analysis software based on the CT volume data, it was possible to measure without these potential sources of error. We believe that our method is more accurate than previous studies.

Age and progression of LB also have been reported in several studies, and the results of this study confirm these reports [1,20,21]. There are several reports that suggest a correlation between height and femoral bowing, and these results are consistent with the present study [1,16]. It is thought that the femoral diaphysis is subjected to bending forces under loading, which results in LB by bone remodeling. We think that progression of LB may lead to decreased height.

On the other hand, we cannot ignore the factor that decreased height with aging may be due to the progressive effects of vertebral fractures, spinal kyphosis, degenerative scoliosis, and shortening of the lower limbs caused by knee osteoarthritis. Aging, short stature, and LB are each intricately involved, and it is difficult to mention the causal relationship while there were correlations in our study.

The correlation between LB of the femur and BMD has been reported by several studies [1,12,22]. The present study showed a weak negative correlation between LB and trochanteric BMD, supporting the idea that the lower the femoral BMD the greater the LB. When the axial pressure on the femur is converted into lateral tension, it is suggested that femurs with low bone density and low bone strength have less mechanical resistance and are more susceptible to remodeling. Basic research to prove this will be necessary in the future. The lumbar spine tends to have larger errors in bone density measurements than the femur due to degenerative scoliosis, osteoarthritis, and calcification of blood vessels. This may be one reason that there was no significant correlation between lumbar BMD and LB. Based on the results of this study, treatment strategies can be planned separately for lateral bowing and anterior bowing in the future.

There are several limitations to this study. First, information about the diagnosis and severity of osteoporosis or osteoarthritis was not available for these patients, nor were details about any treatments. Second, the activity and exercise habits of the patients were unknown. Third, not all facilities have preoperative measurement software and complete CT volume data for the entire length of the femur on the healthy side.

Each point used to define the reference axis can be defined as a single point in three dimensions. Therefore, intra-rater reliability and inter-rater reliability are expected to be small. However, we are also planning to investigate them in detail in the next paper as this is a new method of evaluation.

## Conclusions

In conclusion, we examined 61 patients in detail the factors associated with AB and LB using three-dimensional preoperative measurement software. The results show LB to be weakly correlated with age, height, and femoral bone density, whereas AB did not correlate with any of the patient parameters. In this study, a novel three-dimensional approach has been used for measurements that may be more accurate than plain two-dimensional radiographs.

## References

[1] Shimosawa H, Nagura T, Harato K, Kobayashi S, Nakamura M, Matsumoto M, Niki Y (2019). Variation of three-dimensional femoral bowing and its relation to physical status and bone mineral density: a study with CT. Surg Radiol Anat.

[2] Egol KA, Chang EY, Cvitkovic J, Kummer FJ, Koval KJ (2004). Mismatch of current intramedullary nails with the anterior bow of the femur. J Orthop Trauma.

[3] Park YC, Song HK, Zheng XL, Yang KH (2017). Intramedullary nailing for atypical femoral fracture with excessive anterolateral bowing. J Bone Joint Surg Am.

[4] Nejima S, Kumagai K, Kobayashi H (2021). Coronal shaft bowing of the femur affects varus inclination of the surgical transepicondylar axis in varus knee osteoarthritis. Knee Surg Sports Traumatol Arthrosc.

[5] Kim JM, Hong SH, Kim JM, Lee BS, Kim DE, Kim KA, Bin SI (2015). Femoral shaft bowing in the coronal plane has more significant effect on the coronal alignment of TKA than proximal or distal variations of femoral shape. Knee Surg Sports Traumatol Arthrosc.

[6] Abdelaal AH, Yamamoto N, Hayashi K (2016). Radiological assessment of the femoral bowing in Japanese population. SICOT J.

[7] Im GI, Kwon OJ, Kim CH (2014). The relationship between osteoarthritis of the knee and bone mineral density of proximal femur: a cross-sectional study from a Korean population in women. Clin Orthop Surg.

[8] Lasam MP, Lee KJ, Chang CB, Kang YG, Kim TK (2013). Femoral lateral bowing and varus condylar orientation are prevalent and affect axial alignment of TKA in Koreans. Clin Orthop Relat Res.

[9] Sebastian AS, Wilke BK, Taunton MJ, Trousdale RT (2014). Femoral bow predicts postoperative malalignment in revision total knee arthroplasty. J Arthroplasty.

[10] Mullaji AB, Marawar SV, Mittal V (2009). A comparison of coronal plane axial femoral relationships in Asian patients with varus osteoarthritic knees and healthy knees. J Arthroplasty.

[11] Zhang JZ, Zhao K, Li JY, Zhu YB, Zhang YZ (2020). Age-related dynamic deformation of the femoral shaft and associated osteoporotic factors: a retrospective study in Chinese adults. Arch Osteoporos.

[12] Papaioannou I, Pantazidou G, Baikousis A, Korovessis P (2020). Femoral bowing and femoral neck-shaft angle evaluation can reduce atypical femoral fractures in osteoporotic patients: a scientific report. Cureus.

[13] Soh HH, Chua IT, Kwek EB (2015). Atypical fractures of the femur: effect of anterolateral bowing of the femur on fracture location. Arch Orthop Trauma Surg.

[14] Dell RM, Adams AL, Greene DF (2012). Incidence of atypical nontraumatic diaphyseal fractures of the femur. J Bone Miner Res.

[15] Oh Y, Fujita K, Wakabayashi Y, Kurosa Y, Okawa A (2017). Location of atypical femoral fracture can be determined by tensile stress distribution influenced by femoral bowing and neck-shaft angle: a CT-based nonlinear finite element analysis model for the assessment of femoral shaft loading stress. Injury.

[16] Oh Y, Wakabayashi Y, Kurosa Y, Ishizuki M, Okawa A (2014). Stress fracture of the bowed femoral shaft is another cause of atypical femoral fracture in elderly Japanese: a case series. J Orthop Sci.

[17] Lee YK, Yeom J, Jang BW, Nho JH, Suh YS, Koo KH (2019). Reliability of measuring lateral bowing angle of the femur in patients with atypical femur fractures. J Orthop Surg (Hong Kong).

[18] Liaw CK, Chen YP, Wu TY, Fuh CS, Chang RF (2019). New computerized method in measuring the sagittal bowing of femur from plain radiograph-a validation study. J Clin Med.

[19] Higuma Y, Tomari K, Noguchi T, Ichimura R (2016). Evaluation of the Anatomical and Mechanical Axis of the Femur in the Sagittal Plane by Three-Dimensional Imaging. Jpn. J. Joint Dis.

[20] Maratt J, Schilling PL, Holcombe S, Dougherty R, Murphy R, Wang SC, Goulet JA (2014). Variation in the femoral bow: a novel high-throughput analysis of 3922 femurs on cross-sectional imaging. J Orthop Trauma.

[21] Demes B (2007). In vivo bone strain and bone functional adaptation. Am J Phys Anthropol.

[22] Feola M, Rao C, Tempesta V, Gasbarra E, Tarantino U (2015). Femoral cortical index: an indicator of poor bone quality in patient with hip fracture. Aging Clin Exp Res.

